# Aboveground and Belowground Insect Herbivory Changes Maize‐Wireworm Interactions via Root Volatile Cues

**DOI:** 10.1111/pce.70578

**Published:** 2026-04-29

**Authors:** Diego M. Magalhães, Gareth A. Thomas, Oliver Clark‐Hattingh, Anusha Mohan‐Kumar, Pradip Songara, John C. Caulfield, David M. Withall, Michael A. Birkett, József Vuts

**Affiliations:** ^1^ Rothamsted Research Harpenden UK

**Keywords:** attractant blend, belowground plant‐insect interaction, field experiment, herbivore‐induced plant volatiles, rhizosphere, root volatiles, small lipophilic molecules, soil chemical ecology

## Abstract

The rhizosphere harbours critical plant‐herbivore interactions often mediated by small lipophilic molecules (SLMs). Despite the agricultural importance of many soil‐dwelling insects, surprisingly little is known about chemically mediated belowground crop‐pest interactions. Root and shoot herbivores can influence these interactions by modifying the chemistry of their shared host, thereby influencing interactions with other organisms across trophic levels. We combined laboratory, semi‐field and field‐based experiments to determine whether single and simultaneous above and belowground herbivory affects the olfactory orientation of soil‐dwelling *Agriotes* wireworms towards maize roots. Different herbivore combinations produced distinct SLM profiles, and wireworms were most significantly attracted to the roots of plants under dual herbivory (wireworms + *Ostrinia nubilalis* larvae on leaves). A synthetic SLM blend, characteristic of dual herbivory, was significantly attractive in semi‐field and field environments. These findings highlight the ecological significance of systematically induced SLMs in shaping interactions between a generalist belowground pest and its host plant.

## Summary statement

Maize plants under shoot, root and dual herbivory produce distinct root small lipophilic molecule (SLM) profiles. Soil‐dwelling wireworms, which are serious crop pests, behaviourally preferred a synthetic SLM blend identified from maize under dual herbivory in lab, semi‐field and field assays.

## 1 Introduction

Plants release volatile organic compounds (VOCs) in response to insect herbivory, known as herbivore‐induced plant volatiles (HIPVs) (Turlings and Erb 2018). These compounds can be emitted locally at the site of damage or systemically from undamaged tissues (Heil and Ton 2008; Kessler and Mueller 2024). HIPV semiochemicals mediate interactions between different trophic levels by (1) repelling or attracting herbivores (Guerrieri and Rasmann 2024); (2) attracting herbivore natural enemies (Kessler and Baldwin 2001; Thompson et al. 2026); (3) interfering with the attraction of pollinators (Kessler et al. 2011; Rusman et al. 2019) or (4) priming defence responses in neighbouring plants (Engelberth et al. 2004; Kessler et al. 2023).

Roots release distinct blends of diffusible small lipophilic molecules (SLMs) in response to insect herbivory. SLMs partly overlap with HIPVs but encompass a broader chemical class that includes volatile, semi‐volatile and potentially nonvolatile constituents (Hiltpold et al. 2013). Because many SLMs have low vapour pressure, they are unlikely to function as long‐distance airborne signals and instead act over shorter spatial scales, diffusing through soil pore spaces or occurring as components of root exudates that generate local chemical gradients in the rhizosphere. Although carbon dioxide (CO_2_) was initially considered the main signal guiding root‐feeding herbivorous arthropods, its high environmental variability limits its reliability (Johnson and Nielsen 2012), and current evidence supports a hierarchical cue system, in which CO_2_ functions at long range, while plant‐derived SLMs mediate precise, short‐range host location (Arce et al. 2021; Erb et al. 2013), a strategy also exploited by herbivore natural enemies (Ali et al. 2011; Rasmann et al. 2005). Such a hierarchy implies that both volatile and less volatile SLMs contribute to host‐finding behaviour, depending on their mobility in soil and the sensory range of the responding organism. Given this chemical and behavioural complexity, a more precise description of underlying mechanisms, such as distinguishing between kinetic and tactic responses (Miller et al. 2009), is essential for understanding plant‐herbivore interactions in the rhizosphere.

Root and shoot herbivores can influence each other indirectly by modifying the chemistry of the shared host plant, consequently altering the plant's above and belowground interactions with organisms across trophic levels. Specific HIPVs released from herbivory by a single species often differ from those produced under simultaneous attack by multiple species (Clavijo McCormick et al. 2012), even when spatially separated tissues such as roots and shoots are attacked. Simultaneous root and shoot feeding can reduce the attractiveness of plants to natural enemies both above and belowground (Pierre et al. 2011; Rasmann and Turlings 2007), although outcomes vary among taxa. For instance, after 1 week of aphid feeding, predatory ladybirds preferred dually infested maize plants over singly infested ones, whereas parasitoids showed no such preference (Hauri et al. 2025). Shoot feeding can induce systemic quantitative and qualitative changes in root SLM production, reducing the plant's attractiveness to soil‐dwelling herbivores (Robert et al. 2012). The same is true for root‐feeding (Anderson et al. 2011; Nardi et al. 2023), enhancing the attraction of aboveground natural enemies (Johnson et al. 2013; Wäckers and Bezemer 2003) and pollinators (Barber et al. 2011; Poveda et al. 2003). These represent the complex and context‐dependent nature of root‐shoot signalling (A'Bear et al. 2014; van Dam and Heil 2011).

In this study, we combined laboratory, semi‐field and field behavioural assays with coupled gas chromatography‐electroantennography (GC‐EAG) and gas chromatography‐mass spectrometry (GC‐MS) to determine if single and simultaneous above and belowground herbivory affects the orientation behaviour of soil‐dwelling wireworms (*Agriotes* spp., Coleoptera: Elateridae), generalist root‐feeding larvae that cause significant damage to major crops (Furlan 2005). We used maize (*Zea mays* L., Poaceae) as the host plant and caterpillars of the European corn borer (*Ostrinia nubilalis* Hübner) (Lepidoptera: Pyralidae) as a generalist shoot herbivore. Finally, we evaluated behavioural responses of wireworms to identified root SLM blends under field conditions.

## 2 Materials and Methods

### 2.1 Plants

Maize seeds (cv. Delprim; Delley Samen und Pflanzen AG, Switzerland) were sown individually in plastic pots (7 × 10 cm diameter) with fertilised soil (Rothamsted prescription mixture: pH 5.5–6.0; 75% medium‐grade peat, 12% sterilised loam, 3% medium‐grade vermiculite, 10% 5 mm lime‐free grit) and placed in an insect‐free greenhouse (23 ± 1°C, 60 ± 10% RH, 15 L:9D). Delprim is a commercially available hybrid with high agronomic relevance and well‐documented experimental robustness; it was selected based on its established effects on the olfactory orientation of belowground organisms (e.g., Rasmann et al. 2005; Robert et al. 2012). Plants used for the experiments were 12‐day‐old and had four fully developed leaves (BBCH 14), as wireworms prefer to damage young seedlings (Poggi et al. 2021). Twenty‐four hours before the experiments, plant roots were gently washed with distilled water, and plants were transplanted either to glass vessels for VOC collection or to olfactometer pots (see ‘Olfactometer behavioural assays’).

### 2.2 Insects

Wireworms were collected from the verge of a nonpesticide‐sprayed wheat field at Rothamsted Research, Harpenden, United Kingdom, as well as from other UK farm sites (Supporting Information: Table S1). Species composition, determined using morphological characteristics (Furlan et al. 2021), was as follows: *Agriotes obscurus* 55%, *A. lineatus* 32%, *A. sputator* 7%, *Agriotes* spp. 6% (data not shown). Larvae were kept under controlled laboratory conditions (23 ± 1°C, 60 ± 10% RH, 15 L:9D) in 50 mL Greiner centrifuge tubes (Merck, UK) filled with sand sterilised at 172°C for 48 h and adjusted to 10% moisture (v/v). Wireworms were fed maize seedlings, and individuals measuring at least 10 mm in length were transferred to moist sand (10% v/v) without food 24 h before sampling and behavioural assays.

*Ostrinia nubilalis* eggs were obtained from HUN‐REN ATK Plant Protection Institute, Hungary. Hatching larvae were kept at Rothamsted Research on an artificial diet (chickpea flour, yeast, Wesson's salt mix, methylparaben, sorbic acid, ascorbic acid, streptomycin sulphate and carbendazim) under the same controlled conditions as wireworms. Second‐instar caterpillars were used in the experiments, representing the first annual generation of *O. nubilalis* developing on young maize plants (Spangler and Calvin 2001). Before infestation, larvae were starved for 6 h to induce immediate feeding damage.

### 2.3 Collection of Chemicals

i. Aboveground VOC collection: For dynamic headspace sampling (air entrainment) of maize aboveground tissues (leaves, stem), plants were randomly assigned to UD, wireworm‐damaged (WD), *O. nubilalis*‐damaged (OND) and wireworm + *O. nubilalis‐*damaged (WOND) treatments. Each infested plant received either five wireworms (WD), five second‐instar *O. nubilalis* (OND) or both (WOND). Two 12‐day‐old plants were enclosed in a glass vessel (30 × 12 cm ID), open at the bottom and closed at the top except for an inlet and outlet port. The bottom was closed with two semi‐circular aluminium plates (12 cm diam.) with a hole in the centre to accommodate the stem. Charcoal‐filtered air was pumped in at 1 L/min and drawn out at 0.6 L/min through a glass tube holding 50 mg Porapak Q polymer (50/80 mesh; Supelco, Bellefonte, PA, USA) sandwiched between glass wool plugs. Before collections, each Porapak Q tube was rinsed with 4 mL freshly distilled diethyl ether and heated under a continuous nitrogen flow at 132°C for 2 h to remove contaminants. VOCs were collected for 96 h (*n* = 4/treatment). Trapped VOCs were eluted from the Porapak Q polymer with 750 µL of freshly distilled diethyl ether, concentrated to 50 µL under a gentle nitrogen stream and stored at –20°C until use.
ii. Belowground SLM collection: Root material from UD, WD, OND and WOND maize plants was washed in distilled water, flash‐frozen in liquid nitrogen and pulverised to a fine powder using a pestle and mortar. Root powder (0.4 g) was then transferred to a glass vial with a septum lid. A 100 µm polydimethylsiloxane solid‐phase microextraction (SPME) fibre (Supelco, Bellefonte, USA) was inserted through the septum and left exposed to the headspace for 60 min at 40°C (*n* = 4/treatment) (Thomas et al. 2025).
iii. To determine diffusion of components of the WOND blend (Table 1) in the sand‐filled olfactometer (‘Olfactometer behavioural assays’), 40 µL blend in diethyl ether solvent was applied to a piece of filter paper, which was placed in a side chamber. After allowing the solvent to evaporate completely within 60 s, SLMs were collected for 24 h on 2.5 cm pieces of PDMS tubing (VWR International, UK) placed at 0, 6 and 12.5 cm (= central chamber) from the source along the olfactometer arm (*n* = 4/distance), similarly to Schulz‐Bohm et al. (2018). PDMS tubes were sealed into glass ampoules under nitrogen and stored at –20°C until GC analysis (see ‘Gas chromatography‐flame ionisation detector (GC‐FID) analysis’). Before the experiments, PDMS tubing was soaked in 100% methanol for 24 h, then heated at 180°C under a constant stream of nitrogen for 1.5 h (Vuts et al. 2020).

**Table 1:** Composition (µg/µL) of synthetic blends used in olfactometer bioassays. The ratio of constituents was determined by GC analysis. Maize plant treatments: UD, undamaged, WD, wireworm‐damaged, WOND, wireworm and *Ostrinia nubilalis*‐damaged. KI, Kováts retention index values, which are proportionate to boiling points, with lower KI values corresponding to lower boiling points.

Compound	KI	UD	WD	WOND	Six‐component WOND blend
Hexanal	779	1.0	0.6	1.7	1.7
γ‐Butyrolactone	859	0.9	1.2	2.3	0
1‐Octen‐3‐ol	965	2.4	1.6	2.4	0
3‐Octanone	968	0.2	0.4	0.6	0
(*E*)‐2‐Octenal	1035	0.8	0.3	1.2	0
(*E*)‐2‐Octen‐3‐ol	1051	0.1	0	0.3	0
1‐Octanol	1055	0	0	0.1	0
2‐Nonanone	1072	0	0	0.1	0
Geranylacetone	1433	4.3	2.9	4.4	0
(*E*)‐Caryophyllene	1434	0.1	2.6	1.5	1.5
( + )‐δ‐Cadinene	1497	10.8	5.1	8.5	8.5
(*E,E*)‐α‐Farnesene	1498	0	0.5	0.3	0.3
Bisabolene	1541	3.4	0	0.5	0.5
n‐Heptadecane	1699	12.4	13.9	8.7	8.7

### 2.4 Larval Electrophysiology

Electrophysiological responses of wireworm antennae to components of the synthetic WOND SLM blend were recorded using coupled GC‐electrophysiology (GC‐EAG) (Wadhams 1990). Individual larvae were immobilised by chilling on ice, and the head with the antennae removed. Glass capillaries were filled with electrolyte solution (7.55 g/L sodium chloride, 0.64 g/L potassium chloride, 0.22 g/L calcium chloride, 0.86 g/L sodium bicarbonate, 1.73 g/L magnesium chloride, 0.61 g/L sodium orthophosphate). The capillary encasing the Ag/AgCl ground electrode was inserted into the head capsule to the base of an antenna, while a long hair on the distal antennal segment was inserted into the capillary with the recording electrode. Antennal signals were amplified using an UN‐06 high‐impedance amplifier (Ockenfels Syntech GmbH, Germany). Separation of blend constituents was achieved on an Agilent 6890 N GC (Agilent Technologies), equipped with a cool on‐column (COC) injector and a flame ionisation detector (FID). The column was HP‐1 (50 × 0.32 mm ID, 0.52 μm film thickness, J & W Scientific, USA). The oven temperature was maintained at 30°C for 2 min, then programmed at 5°C/min to 250°C. The carrier gas was helium. The outputs from the EAG amplifier and the FID were monitored simultaneously and analysed using a customised software package (Syntech GC/EAD for Windows v 2.3 09/1997). One μL aliquots of the synthetic WOND blend (prepared as described below) were injected. A compound was considered EAG‐active if it evoked a consistent antennal response in at least three coupled runs.

### 2.5 GC‐FID Analysis

i. Aboveground VOC extracts: Headspace samples collected from aboveground maize tissues were analysed using an Agilent 6890 N GC (Agilent Technologies) equipped with a COC injector, an FID and a nonpolar HP‐1 column. The oven temperature was maintained at 30°C for 1 min, programmed to increase at 5°C/min to 150°C, held for 0.1 min, then programmed to increase at 10°C/min to 250°C, and held for 20 min. The carrier gas was hydrogen. As an internal standard, 1 μL of octane was added to each sample with a final concentration of 9.8 μg/mL for quantification. One μL aliquots were injected. Data were collected with GC Open Lab.
ii. Belowground SLM extracts: Compounds absorbed on SPME fibres were analysed on an Agilent 6890 N GC equipped with a thermal desorption injector, an FID and a nonpolar HP‐1 column by manually inserting the fibre into the OPTIC PTV unit of the GC (30 → 250°C ballistically at 16°C/s). The GC conditions were as above. Approximate quantification of each tentatively identified compound was performed externally by injecting 10 ng authentic standard in a 1 µL aliquot and amounts standardised by root fresh weight.
iii. SLM diffusion: PDMS pieces were put individually into empty glass Tenax tubes (Sigma‐Aldrich, Gillingham, UK), which were inserted into the OPTIC PTV unit of the GC.

### 2.6 Coupled GC‐Mass Spectrometry (GC‐MS) Analysis

Peaks in VOC and SLM samples were tentatively identified by comparing their mass spectra and Kováts retention index (KI) values with those in the NIST11 mass spectral database. A Micromass Autospec Ultima magnetic sector mass spectrometer (Waters, Milford, MA) attached to an Agilent 6890 N GC was used. The GC was fitted with an HP‐1 column and equipped with a COC injector for VOC samples (1 μL aliquots) and a thermal injector port for SLM samples. Ionisation was by electron impact (70 eV, 220°C). The GC oven temperature was maintained at 30°C for 5 min and then programmed to increase at 5°C/min to 250°C. Helium was the carrier gas. Tentative peak identities in SLM samples were confirmed via comparison of their KI values, calculated using a series of C_7_–C_22_ alkanes, and mass spectra with those of authentic standards run under the same conditions.

### 2.7 Chemicals

Authentic standards were purchased from Sigma‐Aldrich, UK: γ‐butyrolactone (99%), 3‐octanone (98%), 1‐octanol (99%), 2‐nonanone (99%), geranylacetone (97%), n‐heptadecane (99%), (*E*)‐caryophyllene (98%); from Alfa Aesar, UK: (*RS*)‐1‐octen‐3‐ol (98%), bisabolene (mixture of isomers); from Fluka, Switzerland: hexanal (98%); from Acros Organics, UK: (*E*)‐2‐octenal (95%); and from BOC Sciences, USA: (+ )‐δ‐cadinene (50%). (*E*)‐2‐octen‐3‐ol (Avocado Research Chemicals, UK). (*E,E*)‐α‐Farnesene was synthesised (Hassemer et al. 2016). Hexanal and (*E*)‐2‐octenal were purified by short‐path distillation immediately before use.

### 2.8 Olfactometer Behavioural Assays

Wireworm behavioural responses to maize root SLMs were assessed using a four‐arm olfactometer (Supporting Information: Figure S1A). The olfactometer consisted of a central acrylic chamber (10 × 8 cm ID) attached to four side arms (12 × 3 cm ID), each leading to an acrylic treatment pot (10 × 8 cm ID) (Small‐Life Supplies, UK). At the junction between each side arm and treatment pot, a fine metal mesh prevented wireworms from reaching the pot and feeding on roots. A second, cone‐shaped metal mesh allowed wireworms to enter the side arms from the central chamber through a ca. 4‐mm narrowing but prevented their return. All mesh components were rinsed with acetone and baked at 150°C for at least 2 h before use.

The olfactometer was filled with sand that had been oven‐baked at 172°C for 48 h and adjusted to 10% (v/v) moisture. For plant‐based assays, 12‐day‐old maize plants were transferred into the sand‐filled treatment pots 24 h before the bioassays to allow acclimation. Ten wireworms were released into the central chamber and allowed to move freely for 24 h, after which the olfactometer was disassembled, and the number of wireworms within each side arm was registered. Responders were larvae that entered any olfactometer arm within 24 h, while individuals remaining in the central chamber were classified as nonresponders and excluded from proportional choice calculations. *N* = 8/experiment, except UD‐WOND *n* = 4, WOND blend *n* = 10, using fresh plants and wireworms for each replicate run simultaneously. While consistent trends were observed across treatments, limited replication for some contrasts (UD vs. WOND) reduces statistical power, and these results should therefore be interpreted cautiously. Before each experiment, the olfactometer was washed with soap, rinsed with distilled water and allowed to air‐dry.

The following experiments were performed:

*Setup 1: Control chambers (Experiment 1)*. All four treatment pots were filled with moistened sand only to assess wireworm movement in an odour‐free environment.

*Setup 2: Live wireworms (Experiment 2)*. Identical to setup 1, except that five fed wireworms were placed in one treatment pot to assess potential attraction to conspecifics.

*Setup 3: Individual plant treatments (Experiments 3–6)*. Three treatment pots contained moistened sand (control), and the fourth contained a plant subject to one of four treatments (UD, WD, OND, WOND). WD and OND plants were exposed to five wireworms or five second‐instar *O. nubilalis* caterpillars, respectively, for 24 h before testing. WOND plants were subjected to simultaneous shoot and root herbivory for 24 h.

*Setup 4: UN versus damaged plants (Experiments 7–9)*. Two opposing treatment pots contained a UD plant and either a WD, OND or WOND plant. The remaining two pots contained moist sand controls.

*Setup 5: Damaged plant comparisons (Experiments 10–12)*. Pairs of damaged treatments were tested against each other: OND versus WD, WOND versus WD and WOND versus OND. The two remaining pots contained moist sand controls.

*Setup 6: Synthetic blends of SLMs (Experiments 13–16)*. Synthetic multi‐component blends of compounds identified from maize SLM extracts by GC‐FID and GC‐MS were prepared in redistilled diethyl ether solvent as a carrier (see Table 1 for compositions). Alongside the UD, WD and WOND blends (Experiments 13–15), a six‐component WOND blend was formulated based on wireworm antennal GC‐EAG responses (Experiment 16). A 40 µL aliquot of each synthetic blend was applied onto 2 cm^2^ strips of filter paper (Whatman, UK), solvent allowed to evaporate for 60 s, then buried near the metal mesh in the corresponding treatment pot. Control pots received 40 µL of diethyl ether only.

### 2.9 Semi‐Field Experiments

Semi‐field experiments were conducted to investigate wireworm responses to a simplified, six‐component synthetic WOND blend, as defined via GC‐EAG/olfactometer assays. The experiments were carried out in square trays (80 × 80 cm) filled with 10 L of sand at 10% (v/v) moisture level (Supporting Information: Figure S1B). Four plastic plant pots (6 × 6.5 cm diameter) were placed at the corners of each tray, leaving a ca. 1 cm wide strip of sand between the pot and the tray wall. Each pot had 10 evenly spaced holes (ca. 4 mm diameter) located midway up the pot wall, dimensions selected to permit unrestricted entry of wireworms based on head capsule size (< 1.5 mm) (Furlan et al. 2021). Before the experiment, 40 µL of freshly redistilled diethyl ether was applied to pieces of filter paper, allowed to evaporate and placed at the bottom of three pots (controls). Forty µL of the six‐component WOND blend was similarly applied to a strip of filter paper (composition in µg/µL: hexanal; (*E*)‐caryophyllene; (+)‐δ‐cadinene; (*E,E*)‐α‐farnesene; bisabolene; n‐heptadecane 17:15:85:3:5:87) and placed at the bottom of the fourth pot (treatment). All pots were filled with sand and packed around to avoid air pockets between the pot and the surrounding sand. Twenty wireworms were released at the centre of each tray. After 24 h, all pots were removed, and the number of larvae in each was recorded; each tray was treated as a single experimental replicate (*n* = 10).

Using the same setup, a separate experiment was conducted to determine the temporal dynamics of wireworm movement towards the six‐component WOND blend. Pots were removed at predefined time points (0, 3, 6, 11 and 24 h) to count larvae, then immediately returned to their original positions. This temporal monitoring was intended solely to characterise response patterns over time and was not subjected to inferential statistical analysis across time points; no repeated‐measures comparisons were performed. Each time point, therefore, represents an observational snapshot rather than an independent experimental replicate. All experiments took place in a glasshouse at 23°C:18°C day:night temperatures, under natural light conditions.

### 2.10 Field Experiment

A field trial testing the six‐component synthetic WOND blend was conducted in 2023 at Wild Farm, an organic farm in Radlett near St Albans, UK (Supporting Information: Table S1). One m^2^ plots served as blocks (replicates), with 10 plots tested on 20–27 September and 20 plots tested on 4 October–8 November over a ca. 0.5 ha area of bare, rich organic soil originally supporting grasslands. Plots were arranged parallel to one another 1 m apart, the surrounding area comprising a mix of vegetables and flowers (e.g., cavolo nero, chard, beans, etc.) (Supporting Information: Figure S1C). In each plot, three control and one treatment pot (see ‘Semi‐field experiments’) were installed at the corners randomly across replicates, completely buried at ca. 10 cm depth and covered with soil. Each control pot contained 40 µL of freshly distilled diethyl ether applied to a cotton plug inside a 0.5 mL Eppendorf tube (Starlab, UK), whereas each treatment pot contained the six‐component WOND blend. The solvent was allowed to evaporate, then the lids were closed. Blend composition (μL neat/lure): hexanal, (*E*)‐caryophyllene, (+)‐δ‐cadinene, (*E,E*)‐α‐farnesene, bisabolene and n‐heptadecane 1.7:1.5:8.5:0.3:0.5:8.7. This formulation was determined through a series of preliminary field trials (Supporting Information: Figure S2 with methodology). Lures were replaced weekly throughout the study, and *Agriotes* spp. larvae captured in each trap were collected and recorded. The soil was regularly irrigated throughout our field campaign, with no frosts and generally mild weather conditions. The study area at the site supported relatively large wireworm populations, a baseline seed bait trapping trial catching over 500 *Agriotes* spp. individuals in May–July 2023.

### 2.11 Scanning Electron Microscopy (SEM)

Wireworms were mounted onto aluminium stubs using a 1:1 mixture of Tissue‐Tek and graphite. The samples were rapidly frozen in slushed liquid nitrogen (–207°C) and subsequently transferred to a Quorum cryo‐preparation system. Each sample was etched at –95°C for 5 min and coated with platinum for 60 s at a current of 5 µA. Cryo‐SEM imaging was conducted on the JEOL 6360 LowVac Scanning Electron Microscope operating at a beam current of 5 kV.

### 2.12 Statistical Analysis

To evaluate the influence of all detected compounds (aboveground VOCs and belowground SLMs) on the separation of different plant treatments, principal component analysis (PCA) was applied to the multivariate data. The PCA was performed using a correlation matrix to compare the treatment groups. A generalised linear model (GLM) with deviance analysis, assuming a Gamma distribution and using an inverse as link function, was used to compare the total and individual amounts of volatiles released from different treatments. When significant treatment effects were detected (*p* < 0.05), mean comparisons were conducted using contrast analyses. Wireworm choice behaviour was analysed at the level of the olfactometer replicate. Nonresponding larvae remaining in the central chamber were excluded from the analysis. Counts of responding larvae per arm were analysed using a GLM with a Poisson error distribution and a natural log link function, testing deviations from an equal distribution of responders among arms. For clarity and visual interpretation, results are presented as percentages of responding larvae per replicate in boxplots with overlaid individual data points. Wireworm preference in semi‐field assays was analysed at the tray level using a GLM with binomial error distribution and logit link, comparing the number of larvae entering the WOND treatment pot versus pooled sand control pots per replicate. Trays with no responding larvae were excluded. Overdispersion was evaluated by comparing residual deviance to residual degrees of freedom, and a quasibinomial model was applied where variance inflation was detected. Nontransformed total field catch data/trap were analysed using the Mann–Whitney *U*‐test and pairwise Wilcoxon rank‐sum tests with adjusted *p*‐values (*p* < 0.05). No functional characterisation was done to describe the mechanisms underlying wireworm behaviour. It is unknown whether the observed outcomes in the behavioural assays were caused by kinetic or tactic attraction (Miller et al. 2009); the term ‘preference’ is thus used in the manuscript. All statistical analyses were performed using R Statistical Software (Foundation for Statistical Computing) and GenStat (20th edition; VSN International Ltd, Hemel Hempstead, UK).

## 3 Results

### 3.1 Behaviour Assays With Live Plants

**Figure 1:**
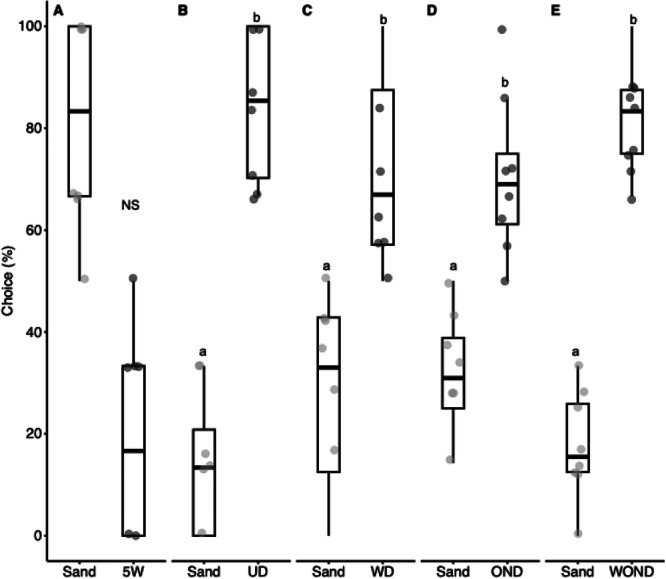
Choices (%) of *Agriotes* spp. wireworms in four‐arm olfactometer assays comparing three sand controls with individual odour sources. (A) Experiment 2: five live wireworms (5 W); (B–E) Experiments 3‐6: undamaged (UD), wireworm‐damaged (WD), *Ostrinia nubilalis*‐damaged (OND) or wireworm‐ and *O. nubilalis*‐damaged (WOND) maize plants. Ten larvae were released per replicate (*n* = 8) and their positions recorded after 24 h. The olfactometer was filled with 10% moist sand. Each point represents the percentage of responding larvae per replicate choosing a given arm; boxplots show the median, interquartile range and 1.5 × IQR whiskers. Letters within each diagram indicate significant differences by Generalised Linear Model (*α* = 0.05). NS, not significant.

In four‐arm olfactometer assays, wireworm responses were calculated as the percentage of individuals entering each arm per replicate. In the control assays containing only sand (Experiment 1), wireworms showed no directional bias, dispersing evenly among the four arms (median choice per arm: arm 1 = 30%, arm 2 = 25%, arm 3 = 20%, arm 4 = 25%; *χ*2 = 0.09, df = 1, *p* = 0.76; total responders = 25%; data not shown). Similarly, no consistent preference was observed when five fed conspecific larvae were used as an odour source (Experiment 2: *χ*2 = 0.001, df = 1, *P* = 1.0; nonresponders = 75%; Figure 1A), with substantial overlap between treatment and sand controls.

When individual plant treatments were tested against sand controls (Experiments 3–6), wireworms consistently showed strong directional responses towards plant‐containing arms: UD plants (*χ*2 = 41.59, df = 1, *p* < 0.001, nonresponders = 36%; Figure 1B), WD (*χ*2 = 27.56, df = 1, *p* < 0.001, nonresponders = 35%; Figure 1C), OND (*χ*2 = 24.84, df = 1, *p* < 0.001, nonresponders = 31%; Figure 1D) or WOND plants (*χ*2 = 37.79, df = 1, *p* < 0.001, nonresponders=34%; Figure 1E) were all preferred over sand, with limited overlap between plant and control distributions. Although statistical significance is reported, the magnitude of these effects is evident from the large separation between medians and interquartile ranges.

**Figure 2:**
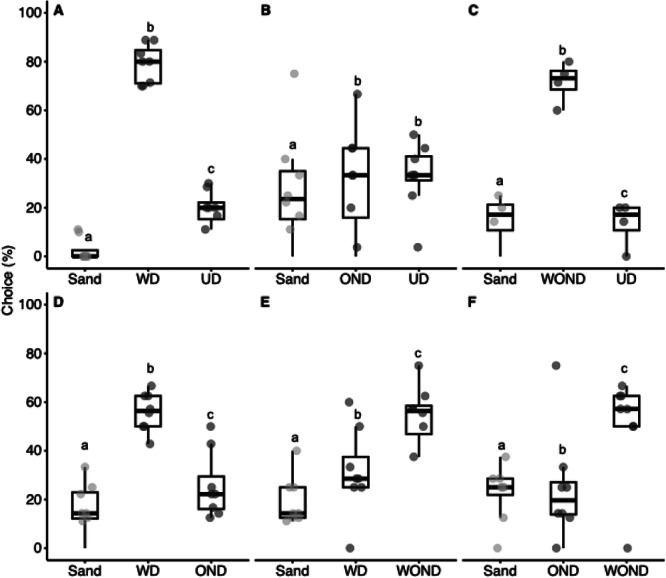
Choices (%) of *Agriotes* spp. wireworms in four‐arm olfactometer assays comparing two sand controls, one undamaged (UD) and one damaged maize plant (wireworm‐damaged [WD], *Ostrinia nubilalis*‐damaged [OND] or wireworm‐ and *O. nubilalis*‐damaged [WOND]). (A–F) Experiments 7–12. Ten larvae were released per replicate (*n* = 8) and their positions recorded after 24 h. The olfactometer was filled with 10% moist sand. Each point represents the percentage of responding larvae per replicate choosing a given arm; boxplots show the median, interquartile range and 1.5 × IQR whiskers. Letters within each diagram indicate significant differences by Generalised Linear Model (GLM) (*α* = 0.05).

Pairwise plant comparisons (Experiments 7–12) revealed graded preferences amongst herbivore treatments (Figure 2). Wireworms preferentially selected WD over UD plants (*χ*2 = 59.66, df = 2, *p* < 0.001, nonresponders = 23%; Figure 2A), and WOND over UD plants (*χ*2 = 17.69, df = 2, *p* < 0.0001, nonresponders = 24%; Figure 2C), whereas responses to UD and OND plants partially overlapped, but both were more preferred than sand control (*χ*2 = 9.24, df = 2, *p* = 0.01, nonresponders = 31%; Figure 2B). WD were preferred over OND plants (*χ*2 = 26.25, df = 2, *p* < 0.001; nonresponders = 28%; Figure 2D). WOND plants elicited the strongest and most consistent responses overall, being more preferred than both WD (*χ*2 = 18.50, df = 2, *p* < 0.001; nonresponders = 25%; Figure 2E) and OND plants (*χ*2 = 23.58, df = 2, *p* < 0.001, nonresponders = 28%; Figure 2F). Median choice towards the preferred treatment typically exceeded 55%–65%, while alternative treatments and sand controls clustered near or below 25%.

### 3.2 Chemical Analysis

**Figure 3:**
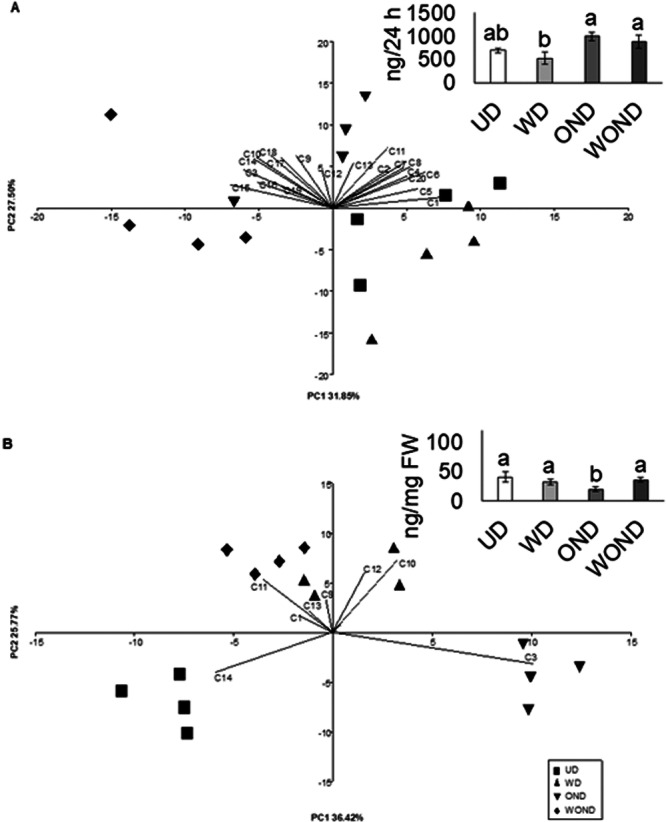
Principal component analysis (PCA) biplots of maize plant volatiles. (A) aboveground headspace VOCs; (B) belowground SLMs from undamaged (UD), wireworm‐damaged (WD), *Ostrinia nubilalis‐*damaged (OND) and wireworm + *O. nubilalis*‐damaged (WOND) maize plants (*n* = 4/treatment). Each biplot shows sample scores along the first and second PC and the contribution of individual compounds to these components. Compound identities are listed in Supporting Information: Tables S2 and S3. Means with the same letter within each insert are not significantly different (*p* > 0.05) by GLM and ANODEV, with further comparisons performed by contrast analyses. Also see Supporting Information: Figure S3 for statistics on individual compounds.

Analyses of headspace samples collected from UD, WD, OND and WOND maize plants revealed both qualitative and quantitative differences in aboveground VOC profiles (GLM *χ*2 = 12.45, df = 3, *p* = 0.005) (Figure 3A, Supporting Information: Table S2). The VOC profile of WD plants closely resembled that of UD plants. In contrast, OND plants emitted significantly higher levels of methyl salicylate, (*E*)‐caryophyllene, α‐bergamotene and (*E*)‐β‐farnesene than UD plants. In the WOND treatment, emissions of α‐pinene, (*E*)‐ocimene and an unknown compound (KI = 1076) were significantly reduced compared to UD plants. PCA showed that the first two components explained 59.35% of the total variance, clearly separating the four treatments based on their chemical profiles. OND and WOND plants formed distinct clusters, whereas UD and WD partially overlapped, with the separation of OND and WOND plants partly associated with higher emissions of (*E*)‐caryophyllene, α‐bergamotene, (*E*)‐β‐farnesene, (*E*)‐4,8‐dimethyl‐1,3,7‐nonatriene and methyl salicylate.

Analyses of belowground SLM profiles revealed significant qualitative and quantitative differences among treatments (GLM *χ*2 = 52.88, df = 3, *p* < 0.001) (Figure 3B, Supporting Information: Table S3, Figure S3). OND plants emitted significantly lower amounts of total SLMs compared with the other treatments. WD plants showed increased levels of (*E*)‐caryophyllene and (*E,E*)‐α‐farnesene, but lower levels of (*E*)‐2‐octenal, (*E*)‐2‐octen‐3‐ol and bisabolene. In contrast, OND plants showed elevated levels of 1‐octen‐3‐ol and reduced levels of geranylacetone, (+ )‐δ*‐*cadinene and n‐heptadecane. The first two PCA components explained 62.19% of the total variance, separating the treatments into three groups: UD, OND, and a combined cluster of WD and WOND. Higher amounts of 1‐octen‐3‐ol separated OND from the other treatments, while increased n‐heptadecane levels characterised UD. (*E*)‐Caryophyllene, (*E,E*)‐α‐farnesene and (+)‐δ*‐*cadinene were more strongly associated with WD and WOND plants.

### 3.3 Behaviour Assays With Synthetic Blends

**Figure 4:**
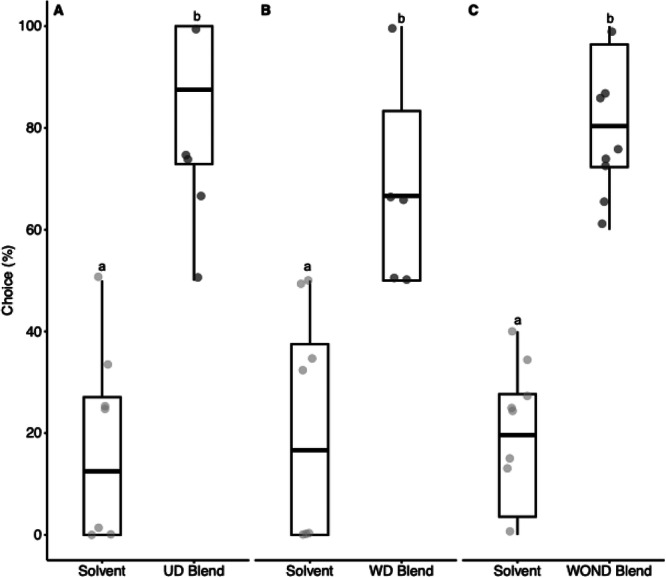
Choices (%) of *Agriotes* spp. wireworms in four‐arm olfactometer assays comparing synthetic SLM blends (UD, WD, WOND) against solvent controls (three arms). (A–C) Experiments 13–15. Blends were formulated based on GC‐FID analyses and applied to filter paper; solvent was allowed to evaporate before testing. Ten larvae were released per replicate (*n* = 8) and their positions recorded after 24 h. The olfactometer was filled with 10% moist sand. Each point represents the percentage of responding larvae per replicate choosing a given arm; boxplots show the median, interquartile range and 1.5 × IQR whiskers. Letters within each diagram indicate significant differences by Generalised Linear Model (GLM) (*α* = 0.05).

Wireworms consistently showed higher proportional choices for arms treated with synthetic SLM blends than for solvent controls (Figure 4). For the UD blend (Experiment 13), the majority of responding larvae selected the treated arm, with median choice values exceeding 80% across replicates, whereas solvent controls typically produced fewer than 20% of responders (*χ*2 = 17.10, df = 1, *p* < 0.001, nonresponders = 69%; Figure 4A). For the WD blend (Experiment 14), wireworms also favoured the synthetic blend over the solvent control (*χ*2 = 3.85, df = 1, *p* = 0.049; nonresponders = 84%, Figure 4B), although responses were more variable among replicates. Median choice values for the WD blend were higher than for solvent controls, but interquartile ranges overlapped more extensively, reflecting weaker and less consistent responses compared to the UD blend. Similarly, the WOND blend (Experiment 15) elicited a clear preference relative to the solvent control (*χ*2 = 6.73, df = 1, *p* = 0.009, nonresponders = 51%; Figure 4C), with median choice values around 75%–80% for the treated arm and substantially lower values for solvent controls.

Dilution assays further showed that attraction to the UD blend persisted at both 250‐fold and 25‐fold dilutions (*χ*2 = 6.20, df = 1, *p* = 0.012 for both). Median choice values remained higher for treated arms than for solvent controls (Supporting Information: Figure S4A,B), indicating that behavioural activity was maintained across a broad concentration range. Diffusion analyses demonstrated that individual SLM constituents of the WOND blend dispersed along the olfactometer arm to different extents (Supporting Information: Figure S5, Table S4), supporting the interpretation that wireworms were exposed to heterogeneous but detectable chemical gradients during behavioural assays.

### 3.4 Coupled GC‐Electroantennography

**Figure 5:**
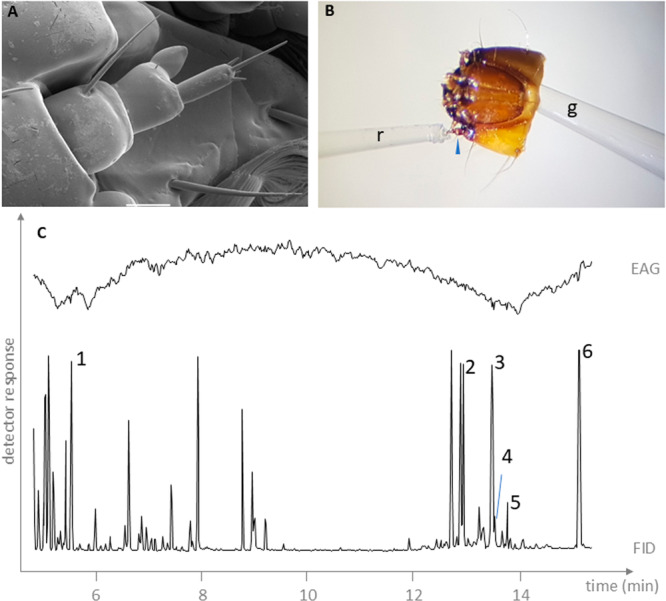
GC‐EAD analysis of the WOND blend. (A) SEM image of the wireworm antenna. (B) head preparation showing the recording electrode (*r*) and the ground electrode (*g*), with antenna marked with an arrowhead. (C) Coupled GC‐EAD trace with bioactive components: (1) hexanal, (2) (*E*)‐caryophyllene, (3). (+)‐δ‐cadinene, (4) (*E,E*)‐α‐farnesene, (5) bisabolene and (6) n‐heptadecane.

Using wireworm antennae (Figure 5A,B) and the behaviourally active WOND blend, six EAG‐active peaks were detected: hexanal, (*E*)‐caryophyllene, ( + )‐δ‐cadinene, (*E,E*)‐α‐farnesene, bisabolene and n‐heptadecane (Figure 5C, Supporting Information: Figure S6).

### 3.5 Olfactometer Assays With the Six‐Component Wond Blend

**Figure 6:**
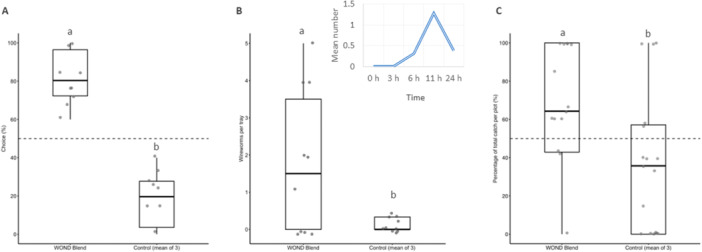
Behavioural responses of *Agriotes* spp. wireworms to the six‐component synthetic WOND blend across different spatial scales. (A) Four‐arm olfactometer assay with one arm containing the WOND blend applied to filter paper and three arms containing sand with a filter paper loaded with solvent control (mean of three control arms) (*n* = 8). Boxplots show the percentage of responding larvae per replicate (nonresponders excluded = 41%), with individual data points overlaid. The dashed line indicates equal distribution (50%). Letters indicate significant differences by GLM (*α* = 0.05). (B) Semi‐field sand tray assays with one treatment pot containing the WOND blend on a filter paper and three pots with sand solvent control (*n* = 12). Boxplots show the number of wireworms per tray. Letters indicate significant differences by GLM (*α* = 0.05). The insert shows the number of wireworms in the treatment pot at intervals over 24 h in a separate experiment. C: Field trial in 1 m^2^ quadrant plots, with one pot containing the WOND blend in an Eppendorf tube and three pots with soil solvent control (*n* = 10 plots). Boxplots show the percentage of total catch per plot, adjusted for three control traps. The dashed line indicates equal allocation (50%). Letters indicate significant differences by Mann–Whitney *U*‐test and pairwise Wilcoxon rank‐sum tests (*α* = 0.05). Also see preliminary field trials in Supporting Information: Figure S2. [Color figure can be viewed at wileyonlinelibrary.com]

Wireworms showed a significant preference for the arm containing the synthetic six‐component blend identified by GC‐EAG, compared with control arms (Experiment 16: *χ*2 = 9.537, df = 1, *p* = 0.002, nonresponders = 41%; Figure 6A).

### 3.6 Semi‐Field Experiments

A binomial GLM at the tray level showed that responding larvae entered the WOND treatment pot more frequently than expected under a random 1:3 allocation between treatment and control pots (log‐odds = 1.50 ± 0.55 SE; *Z* = 4.71; *p* < 0.001; odds ratio = 4.5, 95% CI: 1.68–15.58). Moderate overdispersion was detected (dispersion = 1.98), and when accounted for using a quasibinomial model, the effect was marginal (*t* = 1.89; *p* = 0.096). When analysed relative to the total number of larvae released per tray, entry into the WOND pot remained significant (logit = –2.31 ± 0.25 SE; *Z* = –9.36; *p* < 0.001). Their activity increased over time, reaching a peak approximately 11 h into the 24 h assay period (Figure 6B).

### 3.7 Field Experiment

Significantly more larvae were recovered from pots containing the six‐component synthetic WOND blend, compared to control pots (Mann–Whitney, *p* < 0.001; total catch = 62) (Figure 6C).

## 4 Discussion

Our results show that shoot and root herbivory differentially modify the chemical phenotype of maize roots and that these changes are associated with altered orientation behaviour of soil‐dwelling wireworms. The maize plant functions as an interface between above‐ and belowground environments, conveying the effects of insect feeding to both aboveground HIPV and belowground SLM emissions. Behavioural assays show that wireworms preferentially orient towards herbivore‐induced SLMs relative to those released by UN plants, indicating that SLMs can bias larval movement. Importantly, our data do not demonstrate that root SLMs alone mediate aggregation in natural settings, but rather that they represent one component of the plant‐derived cue complex influencing wireworm host‐location behaviour. While shoot herbivory alone did not alter larval responses compared to UN roots, it systemically enhanced the attractiveness of roots fed upon by conspecific larvae, suggesting interactive effects between above‐ and belowground herbivory on root chemistry. There is thus a complex interplay between above‐ and belowground herbivory that manifests itself in altered root SLM composition, but the underlying biosynthetic mechanisms remain to be elucidated. This complexity is further highlighted by the fact that leaf herbivory in maize does not evoke systemic induction of defence hormones in roots (Erb et al. 2009) but instead suppresses the emission of ethylene, a phytohormone related to root growth (Robert et al. 2012). Our findings support a role for herbivore‐induced root SLMs as short‐range behavioural modifiers within a broader, multi‐cue framework governing wireworm foraging belowground.

The composition of VOCs in shoot headspace extracts in our study, both before and after herbivory, was similar to those previously reported for Delprim maize in response to caterpillar (*Spodoptera* and *Helicoverpa* spp.) feeding, including β‐myrcene, (*Z*)‐3‐hexenyl acetate, linalool, (*E*)‐4,8‐dimethyl‐1,3,7‐nonatriene, geranyl acetate, (*E*)‐caryophyllene, α‐bergamotene, (*E*)‐β‐farnesene, β‐sesquiphellandrene and (*E,E*)‐4,8,12‐trimethyl‐1,3,7,11‐tridecatetraene (De Lange et al. 2020; Erb et al. 2011). Although indole is an essential HIPV that acts as a priming signal in maize (Erb et al. 2015), its induction in response to *O. nubilalis* feeding appears to be genotype‐specific. We did not detect indole emission from Delprim, whereas Turlings et al. (1998) reported its presence in the LG11 variety. On the other hand, wireworm root feeding alone did not alter the shoot VOC profile, similar to *Diabrotica v. virgifera* and *Agriotes* spp. herbivory on maize and ribwort plantain, respectively (Rasmann and Turlings 2007; Wurst et al. 2008).

Dual herbivory on Delprim maize decreased the emission of α‐pinene and (*E*)‐ocimene from leaves compared to plants damaged only by *O. nubilalis*. Although the ecological consequences of such a bouquet shift for aboveground communities are yet to be elucidated, previous studies have shown that root herbivores can strongly influence aboveground interactions (Soler et al. 2005). For example, simultaneous root‐shoot herbivory in maize negatively affects the attraction of third trophic‐level natural enemies (Rasmann and Turlings 2007), while in black mustard, root herbivory by *Delia radicum* reduces shoot (*E*)‐β‐farnesene emission, which again hinders the foraging behaviour of parasitic wasps (Soler et al. 2007). Interestingly, wireworm herbivory has been shown to increase extrafloral nectar production in cotton, suggesting that induced aboveground defences might, in some cases, promote root regrowth and ultimately benefit the herbivores themselves (Wäckers and Bezemer 2003).

Plants under simultaneous above and belowground herbivory often allocate their defences towards the shoot (Rasmann and Turlings 2007). We found that maize exposed to leaf herbivory showed a decreased production of total root SLMs, suggesting that leaves are more inducible than roots, a pattern also reported by Erb et al. (2012). However, this systemic suppression of root emissions contrasts with Rasmann and Turlings (2007) and Robert et al. (2012), who found no such effect of *S. littoralis* leaf herbivory on maize. On the other hand, wireworm‐infested roots released higher amounts of (*E*)‐caryophyllene than UN or *O. nubilalis*‐infested plants, which corroborates earlier studies with *D. v. virgifera* and *S. littoralis* (Rasmann and Turlings 2007; Robert et al. 2012). This sesquiterpene has a well‐documented role in the attraction of entomopathogenic nematodes and is a key defence compound produced by Delprim maize in response to *D. v. virgifera* attack (Hiltpold et al. 2011; Rasmann et al. 2005). Such systemically induced changes in root SLM emissions can thus influence the structure of soil‐dwelling communities, which readily use HIPVs for host location (Ali et al. 2010, 2011). For example, *D. v. virgifera* larvae preferentially orient towards maize roots infested with conspecifics rather than undamaged or leaf‐infested plants (Robert et al. 2012). The outcome of shoot‐root herbivore interactions depends on their arrival sequence (Johnson et al. 2012), with shoot herbivores negatively affecting root herbivores when they attack first, but not when feeding simultaneously. In our study, the PCA revealed that WOND and WD treatments clustered close together, indicating that their root SLM profiles were more similar than those of UD or OND plants, with no significant difference in the total amount of SLMs between WD and WOND. The exact contribution of individual SMLs to wireworm behavioural preferences is still to be evaluated. It should be noted that crushed flash‐frozen roots were used to sample root volatiles; although effective, this method is destructive and does not discriminate between readily emitted and stored compounds.

Wireworms are known to aggregate near host plants in the field (Barsics et al. 2013; Parker and Howard 2001; Salt and Hollick 1946). Our results may suggest that these aggregations are mediated by root SLMs, and not by conspecific odour (la Forgia et al. 2023). Root‐derived aldehydes, for example hexanal, and sesquiterpenes, for example (*E*)‐caryophyllene, have been shown to attract wireworms and other soil‐dwelling herbivores (Johnson and Nielsen 2012; Robert et al. 2012; Barsics et al. 2017; Nikoukar et al. 2025). In our study, the six‐component synthetic WOND blend behaviourally preferred by wireworms contained all these compound classes. We observed that three sesquiterpenes were collected by PDMS tubing placed at the centre of the olfactometer arena, indicating that these compounds may trigger initial orientation, and other components of the blend may influence wireworm behaviour nearer to the SLM source. Interestingly, wireworms still moved towards the synthetic blends in the absence of CO_2_, although to a lesser extent, possibly because the lack of CO_2_ compromised their searching behaviour (Arce et al. 2021).

Rhizosphere microorganisms can also affect plant‐herbivore interactions (Chiriboga et al. 2018; Santos et al. 2014), as insects can use microbial VOCs to locate their hosts (Davis et al. 2013). Additionally, microbes can metabolise, transform or adsorb soil SLMs, thereby acting as a potential sink for bioactive compounds (Schenkel et al. 2018). However, the ecological consequences of simultaneous above and belowground herbivory on these microbial communities remain unclear. By using maize as a model system, we can begin to explore how plant defence mechanisms, mediated by both herbivores and microbes, might influence interactions with higher trophic levels to recruit natural enemies. Identifying the specific SLMs that encode host recognition for wireworms represents an important first step in understanding these complex interactions.

Taken together, our behavioural assays clarify which components of the plant cue are sufficient to bias wireworm movement, while also highlighting the limits of inference from synthetic cues tested in isolation. Both live dual‐infested maize plants and a synthetic SLM blend characteristic of this condition elicited consistent directional responses in wireworms, but these findings derive from complementary and methodologically distinct experiments. Assays with live plants capture the net behavioural outcome of a complex and dynamic plant signal, whereas assays with synthetic blends test whether a defined subset of plant‐derived chemicals is sufficient to elicit orientation responses in the absence of other cues. As a result, the synthetic blend should not be interpreted as a complete proxy for the full suite of plant‐associated cues involved in host selection. Importantly, all behavioural assays involving synthetic SLM blends were conducted in the absence of plants, demonstrating that these compounds are behaviourally active per se but not addressing how they interact with additional plant‐derived cues such as CO₂ gradients from root respiration, soil moisture heterogeneity or other unmeasured metabolites. Host location by wireworms and other soil‐dwelling herbivores is widely understood to involve hierarchical cue use, CO₂ acting primarily at longer distances and plant‐derived SLMs providing short‐range information for host recognition and acceptance (Arce et al. 2021). Because CO₂ was neither measured nor manipulated in the present study, it remains unresolved whether and how the identified SLMs act in combination with CO₂ gradients under natural conditions. Within this broader framework, the behavioural responses to the synthetic blend indicate that SLMs associated with dual herbivory are sufficient to bias wireworm orientation at laboratory, semi‐field and field scales, but do not imply that these compounds alone recapitulate the complete rhizosphere milieu experienced by wireworms. Nonetheless, the consistent behavioural activity of a simplified blend across spatial scales supports its ecological relevance and highlights the potential utility of root SLMs for attractant‐based management strategies.

## Conflicts of Interest

The authors declare no conflicts of Interest.

## Supporting information

Supporting File

## Data Availability

The data that support the findings of this study are openly available in Rothamsted Research at https://doi.org/10.23637/dpwwltaq.
