# Supplementary material for: Aboveground and Belowground Insect Herbivory Changes Maize‐Wireworm Interactions via Root Volatile Cues
Source: Plant Cell Environ. 2026 Apr 29;49(8):5689–702. doi: 10.1111/pce.70578 (PMC13353745; doi:10.1111/pce.70578)
Supplement: Supplementary file 1 — Supporting File [file PCE-49-5689-s001.docx]

Table S1. Coordinates of sites where *Agriotes* spp. wireworms for experimental work were collected.

| Site | Coordinate |
| --- | --- |
| Rothamsted Research | 51.809409, -0.369299 |
| Radlett | 51.69650, -0.30146 |
| Swaffham Prior | 52.293001, 0.244775 |
| Bucksum Farm | 51.770469, -1.016408 |
| Holwell | 51.986075, -0.294207 |

Table S2. Amounts (mean ± SE) of individual compounds of aboveground volatiles emitted by undamaged (UD), wireworm-damaged (WD), *Ostrinia nubilalis-*damaged (OND), and wireworm + *O. nubilalis*-damaged (WOND) maize plants in ng per 24 h.

|  |  | Treatments (ng 24 h^-1^) | | | | | | | | | | | | |
| --- | --- | --- | --- | --- | --- | --- | --- | --- | --- | --- | --- | --- | --- | --- |
| No. | Compounds | KI | UD | | | WD | | | OND | | | WOND | | |
| 1 | α-Pinene | 936 | 33.29 | ± | 14.47 | 44.32 | ± | 9.38 | 39.80 | ± | 11.82 | 11.69 | ± | 5.11 |
| 2 | β-Myrcene | 984 | 28.80 | ± | 11.31 | 16.54 | ± | 10.41 | 19.30 | ± | 5.40 | 13.66 | ± | 2.76 |
| 3 | (*Z*)-3-hexenyl acetate | 988 | 5.92 | ± | 2.97 | 0.00 | ± | 0.00 | 27.75 | ± | 11.67 | 72.78 | ± | 22.39 |
| 4 | Unknown compound | 1008 | 61.20 | ± | 14.03 | 42.67 | ± | 10.47 | 57.41 | ± | 12.65 | 32.37 | ± | 5.44 |
| 5 | Limonene | 1026 | 13.77 | ± | 6.35 | 14.25 | ± | 2.58 | 17.18 | ± | 3.96 | 7.27 | ± | 2.13 |
| 6 | (*E*)-Ocimene | 1041 | 29.31 | ± | 12.91 | 19.41 | ± | 13.62 | 27.05 | ± | 10.60 | 1.05 | ± | 0.73 |
| 7 | Linalool oxide (furanoid) | 1063 | 11.44 | ± | 0.84 | 9.24 | ± | 3.93 | 13.64 | ± | 4.16 | 4.84 | ± | 0.88 |
| 8 | Unknown compound | 1076 | 18.67 | ± | 2.55 | 12.69 | ± | 5.73 | 16.62 | ± | 4.08 | 8.16 | ± | 1.91 |
| 9 | Linalool | 1086 | 287.12 | ± | 12.64 | 169.63 | ± | 56.32 | 285.63 | ± | 35.80 | 290.22 | ± | 78.02 |
| 10 | (*E*)-4,8-dimethyl-1,3,7-nonatriene (DMNT) | 1106 | 13.34 | ± | 3.98 | 5.76 | ± | 0.91 | 31.18 | ± | 5.46 | 31.05 | ± | 3.71 |
| 11 | Benzyl acetate | 1136 | 1.92 | ± | 1.33 | 1.64 | ± | 0.82 | 3.61 | ± | 1.48 | 1.36 | ± | 1.09 |
| 12 | Methyl salicylate | 1175 | 27.89 | ± | 4.82 | 27.71 | ± | 6.14 | 74.36 | ± | 11.81 | 56.27 | ± | 20.98 |
| 13 | Geranyl acetate | 1363 | 45.90 | ± | 8.59 | 52.55 | ± | 22.77 | 67.22 | ± | 5.33 | 46.41 | ± | 8.99 |
| 14 | (*E*)-Caryophyllene | 1438 | 0.84 | ± | 0.97 | 1.14 | ± | 0.63 | 73.90 | ± | 20.14 | 78.22 | ± | 41.19 |
| 15 | α-Bergamotene | 1444 | 21.61 | ± | 8.45 | 6.10 | ± | 2.49 | 45.76 | ± | 5.05 | 81.61 | ± | 22.33 |
| 16 | (*E*)-β-Farnesene | 1450 | 13.87 | ± | 4.10 | 11.81 | ± | 3.00 | 70.43 | ± | 13.32 | 81.22 | ± | 25.24 |
| 17 | β-Sesquiphellandrene | 1459 | 2.47 | ± | 1.11 | 2.14 | ± | 1.44 | 5.27 | ± | 1.33 | 5.39 | ± | 2.70 |
| 18 | Bisabolene | 1510 | 5.07 | ± | 2.05 | 1.70 | ± | 1.14 | 8.03 | ± | 1.32 | 8.49 | ± | 2.28 |
| 19 | (*E,E*)-4,8,12-trimethyl-1,3,7,11-tridecatetraene (TMTT) | 1570 | 7.52 | ± | 2.60 | 1.35 | ± | 1.24 | 8.70 | ± | 3.83 | 7.29 | ± | 2.67 |
| 20 | Unknown compound | 1590 | 60.98 | ± | 15.15 | 86.75 | ± | 36.78 | 106.68 | ± | 15.17 | 47.84 | ± | 10.96 |

Table S3. Amounts (mean±SE) of individual compounds from pulverised maize roots produced by undamaged (UD), wireworm-damaged (WD), *Ostrinia nubilalis-*damaged (OND) and wireworm + *O. nubilalis*-damaged (WOND) maize plants in ng per mg of fresh weight (FW).

|  |  | Treatments (ng mg^-1^ FW) | | | | | | | | | | | | |
| --- | --- | --- | --- | --- | --- | --- | --- | --- | --- | --- | --- | --- | --- | --- |
| No. | Compounds | KI | UD | | | WD | | | OND | | | WOND | | |
| 1 | Hexanal | 779 | 1.10 | ± | 0.23 | 0.63 | ± | 0.37 | 1.08 | ± | 0.26 | 1.89 | ± | 0.21 |
| 2 | γ-Butyrolactone | 859 | 1.04 | ± | 0.61 | 1.33 | ± | 0.16 | 1.38 | ± | 0.25 | 2.61 | ± | 0.31 |
| 3 | 1-Octen-3-ol | 965 | 2.67 | ± | 0.41 | 1.80 | ± | 0.21 | 6.15 | ± | 0.29 | 2.63 | ± | 0.27 |
| 4 | 3-Octanone | 968 | 0.22 | ± | 0.13 | 0.47 | ± | 0.19 | 0.49 | ± | 0.22 | 0.68 | ± | 0.20 |
| 5 | (*E*)-2-Octenal | 1035 | 0.85 | ± | 0.26 | 0.37 | ± | 0.23 | 0.62 | ± | 0.23 | 1.35 | ± | 0.34 |
| 6 | (*E*)-2-Octen-3-ol | 1051 | 0.14 | ± | 0.08 | 0.00 | ± | 0.00 | 0.45 | ± | 0.18 | 0.33 | ± | 0.11 |
| 7 | 1-Octanol | 1055 | 0.00 | ± | 0.00 | 0.00 | ± | 0.00 | 0.00 | ± | 0.00 | 0.13 | ± | 0.08 |
| 8 | 2-Nonanone | 1072 | 0.00 | ± | 0.00 | 0.00 | ± | 0.00 | 0.00 | ± | 0.00 | 0.10 | ± | 0.06 |
| 9 | Geranylacetone | 1433 | 4.79 | ± | 1.62 | 3.19 | ± | 0.57 | 1.28 | ± | 0.48 | 4.89 | ± | 0.84 |
| 10 | (*E*)-Caryophyllene | 1434 | 0.09 | ± | 0.05 | 2.91 | ± | 0.26 | 0.18 | ± | 0.02 | 1.64 | ± | 0.18 |
| 11 | (+)-δ-Cadinene | 1497 | 12.07 | ± | 2.73 | 5.65 | ± | 1.09 | 1.66 | ± | 1.07 | 9.42 | ± | 0.87 |
| 12 | (*E,E*)-α-Farnesene | 1498 | 0.00 | ± | 0.00 | 0.55 | ± | 0.36 | 0.00 | ± | 0.00 | 0.34 | ± | 0.16 |
| 13 | Bisabolene | 1541 | 3.76 | ± | 1.95 | 0.00 | ± | 0.00 | 0.24 | ± | 0.14 | 0.56 | ± | 0.08 |
| 14 | Heptadecane | 1699 | 13.84 | ± | 0.74 | 15.54 | ± | 1.72 | 5.96 | ± | 0.59 | 9.64 | ± | 0.72 |

Table S4. Mean (ng±SE) amount of components of the synthetic WOND blend detected at three distances from the source along the soil olfactometer arm (n=4). Compounds were released from a piece of filter paper placed within a side chamber and captured using short pieces of PDMS

| Compound | Distance (cm) from source | | |
| --- | --- | --- | --- |
|  | 0 | 6 | 12.5 |
| hexanal | 6.14±0.66 | 4.24±0.63 | 2.28±0.18 |
| γ-butyrolactone | 5.88±1.73 | 0.00±0.00 | 0.00±0.00 |
| (*RS*)-1-octen-3-ol | 1031.70±87.57 | 1284.21±94.83 | 285.98±44.78 |
| 3-octanone | 117.42±27.76 | 149.28±39.74 | 113.46±8.00 |
| (*E*)-2-octenal | 618.72±51.36 | 369.50±27.23 | 75.19±12.20 |
| (*E*)-2-octen-3-ol | 478.92±34.23 | 59.11±4.82 | 3.73±2.14 |
| 1-octanol | 443.09±29.44 | 92.88±8.43 | 8.81±0.55 |
| 2-nonanone | 511.46±27.96 | 326.80±13.91 | 166.13±11.56 |
| geranylacetone | 18954.57±5948.77 | 577.85±90.95 | 0.00±0.00 |
| (*E*)-caryophyllene | 17277.41±1909.88 | 3843.42±364.90 | 1885.60±156.08 |
| (+)-δ-cadinene | 8158.14±2211.34 | 1118.24±119.22 | 411.95±37.60 |
| (*E,E*)-α-farnesene | 4184.33±268.54 | 419.73±17.36 | 132.75±9.01 |
| bisabolene | 741.49±138.84 | 0.00±0.00 | 0.00±0.00 |
| n-heptadecane | 20013.61±3219.09 | 165.61±55.21 | 0.00±0.00 |


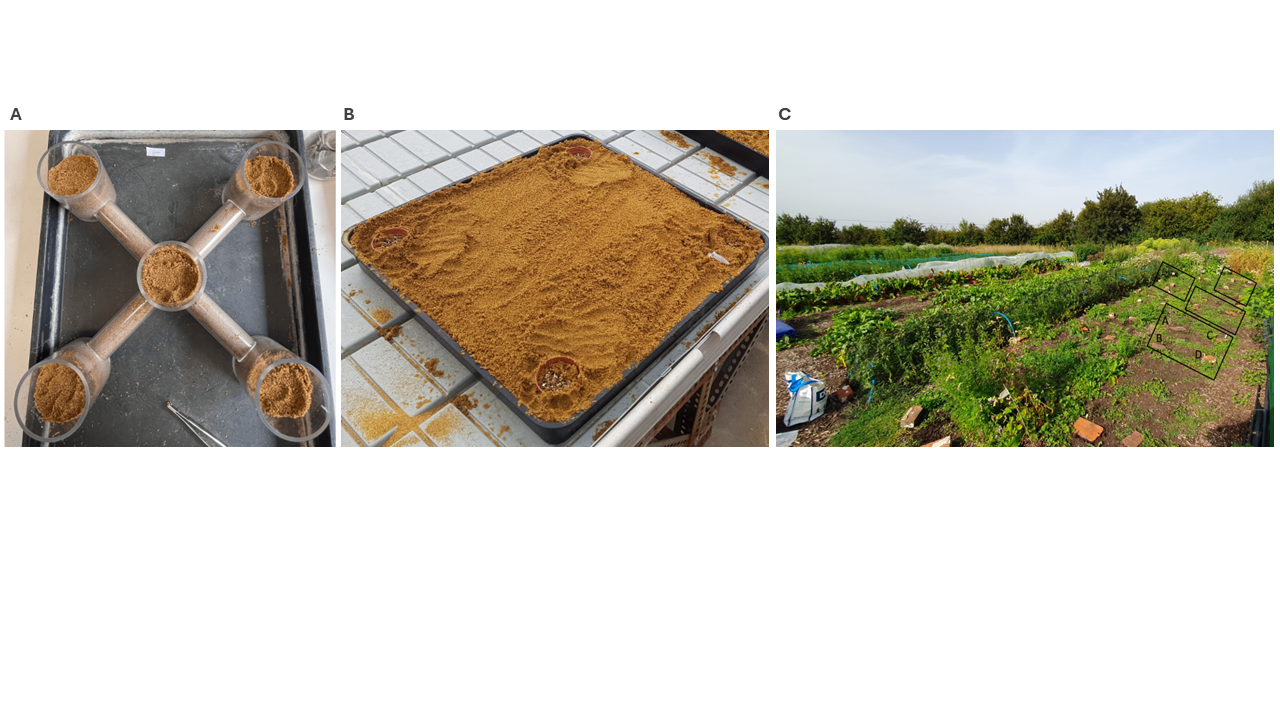


Figure S1. Photo of four-arm olfactometer (A), semi-field experiment arena (B) and field trial (C).


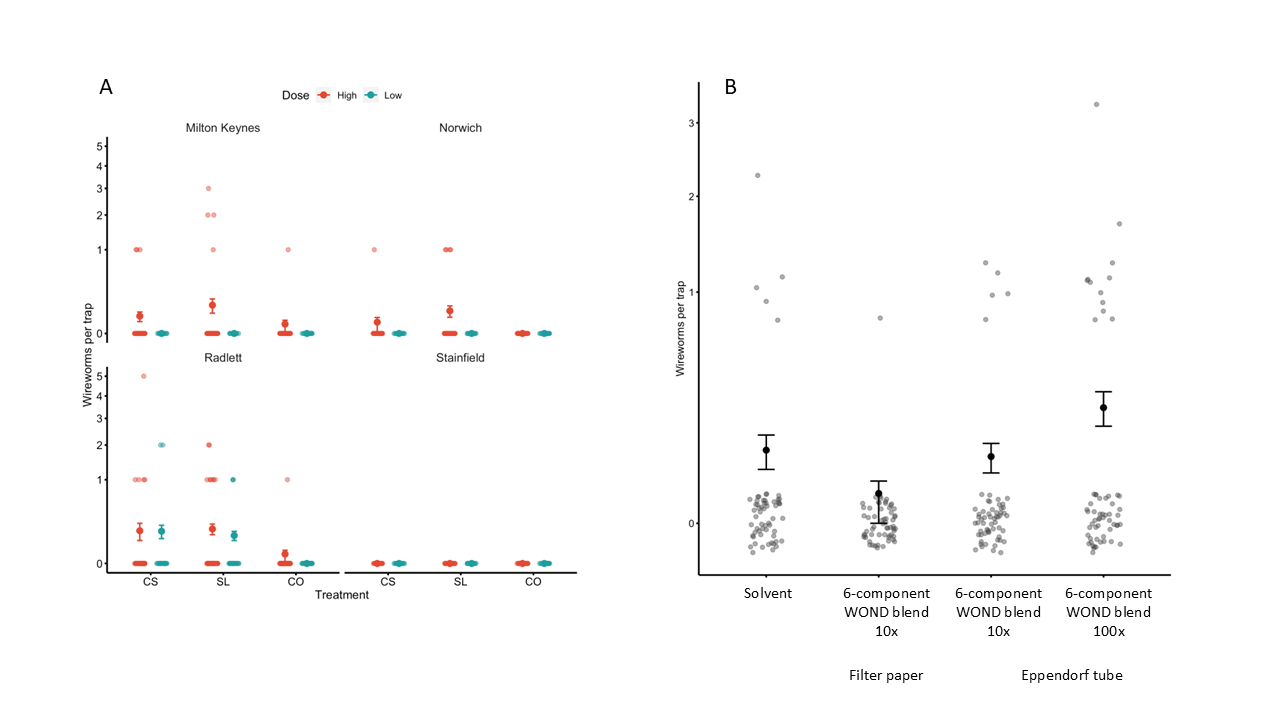


Figure S2. Lure development during preliminary field trials leading to the formulation used for the 6-component WOND blend. A: Field trial at four different UK sites from top left clockwise: Milton Keynes, Norwich, Radlett and Stainfield, using the 6-component WOND blend on pieces of filter paper at the same concentration as in semi-field assays (green bars) and at a 10-fold stronger concentration (red bars) (n=10; see 2.9 for blend details). A soil core sample was taken in the middle of each experimental plot. CS: solvent. SL: 6-component WOND blend. CO: soil core. B: Field trial at the Radlett site using two different formulations and doses: the 6-component WOND blend at a 10-fold stronger dose as in semi-field assays on a piece of filter paper and in an Eppendorf tube with a piece of cotton plug, and also in an Eppendorf tube with a piece of cotton plug at a 100-fold stronger dose as in semi-field assays (n=10). The latter dose was used in the Radlett field experiment (see 2.10 for blend details). Means and SE are plotted with jittered points showing the number of insects caught per trap


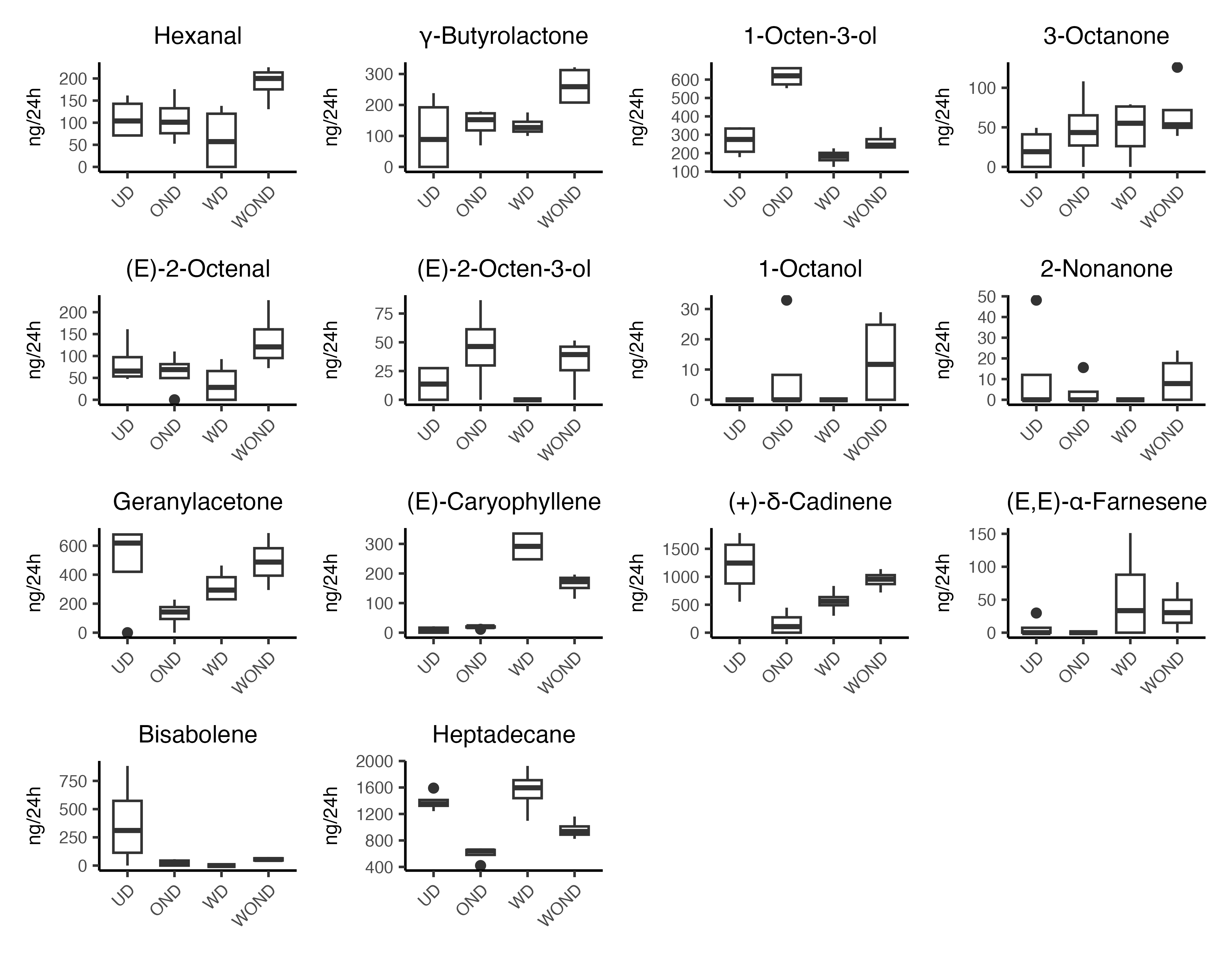


a

a

a

a

ab

a

a

b

b

b

c

a

a

a

a

a

a

a

a

a

a

a

a

b

a

a

a

a

a

a

a

a

c

a

b

bc

a

a

c

b

c

a

ab

bc

a

a

ab

ab

a

b

b

b

b

c

a

b

Figure S3. Quantification of belowground small lipophilic molecules (SLMs) emitted by maize roots. Roots from undamaged (UD), wireworm-damaged (WD), *Ostrinia nubilalis*-damaged (OND), and wireworm + *O. nubilalis*-damaged (WOND) plants were flash-frozen, ground and their headspace sampled by solid-phase microextraction (SPME). Boxes show the median, interquartile range and 1.5× IQR whiskers of the ng SLM/24 h values; points represent individual biological replicates (n=4 per treatment). Different letters above boxplots indicate significant differences among treatments for each compound (GLM, α=0.05). P values: hexanal: 0.089; γ-butyrolactone: 0.005; 1-octen-3-ol: <0.001; 3-octanone: 0.833; (*E)*-2-octenal: 0.896; (*E*)-2-octen-3-ol: 0.041; 1-octanol: 0.147; 2-nonanone: 0.341; geranylacetone: <0.001; (*E*)-caryophyllene: <0.001; (+)-δ-cadinene: 0.003; (*E,E*)-α-farnesene: 0.048; bisabolene: 0.019; heptadecane: <0.001.


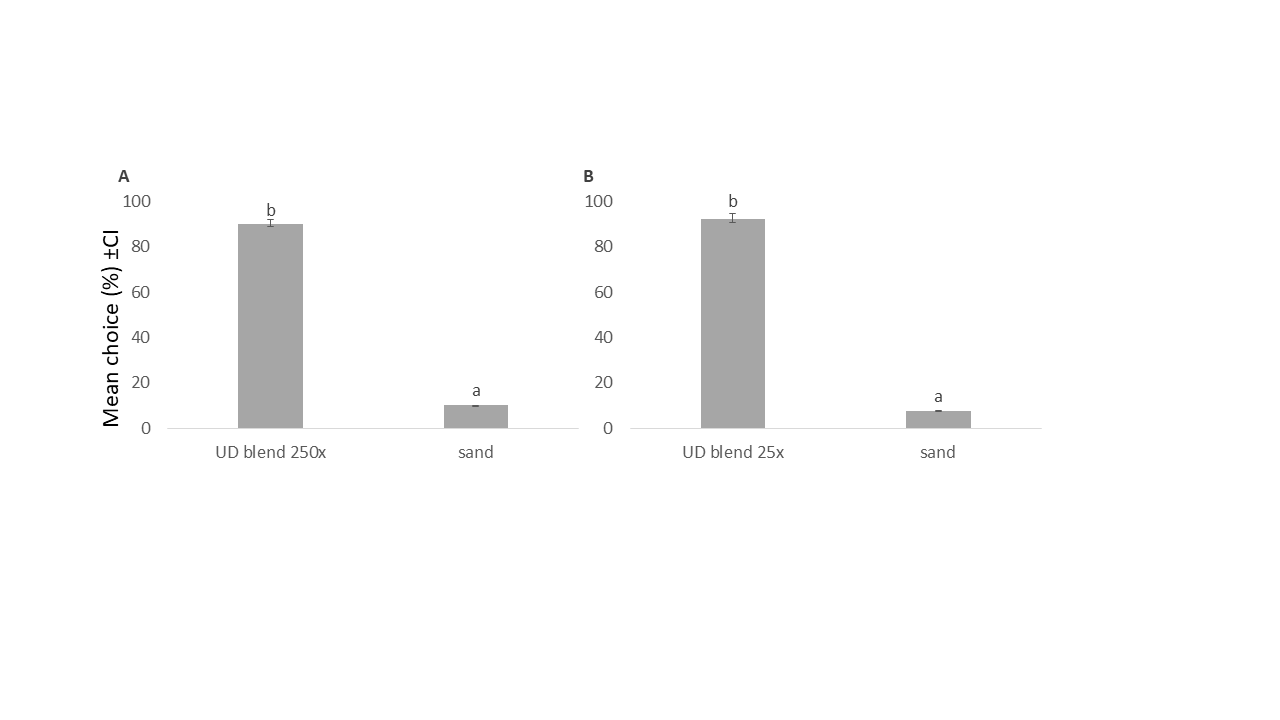


Figure S4. Wireworm responses in a four-arm olfactometer to 250-fold (A) and 25-fold (B) dilutions of the UD synthetic blend vs solvent control (diethyl ether)

Figure S5. Loss of components of a synthetic blend of maize root VOCs, identified from the WOND treatment, along a gradient in the olfactometer arm (n=4). Compounds were applied to filter paper in the side chamber, and their amounts were sampled using short PDMS tubes at the source (0 cm), halfway up the olfactometer arm (6 cm) and in the central chamber (12 cm). Compounds are ordered by increasing molecular weight from left to right


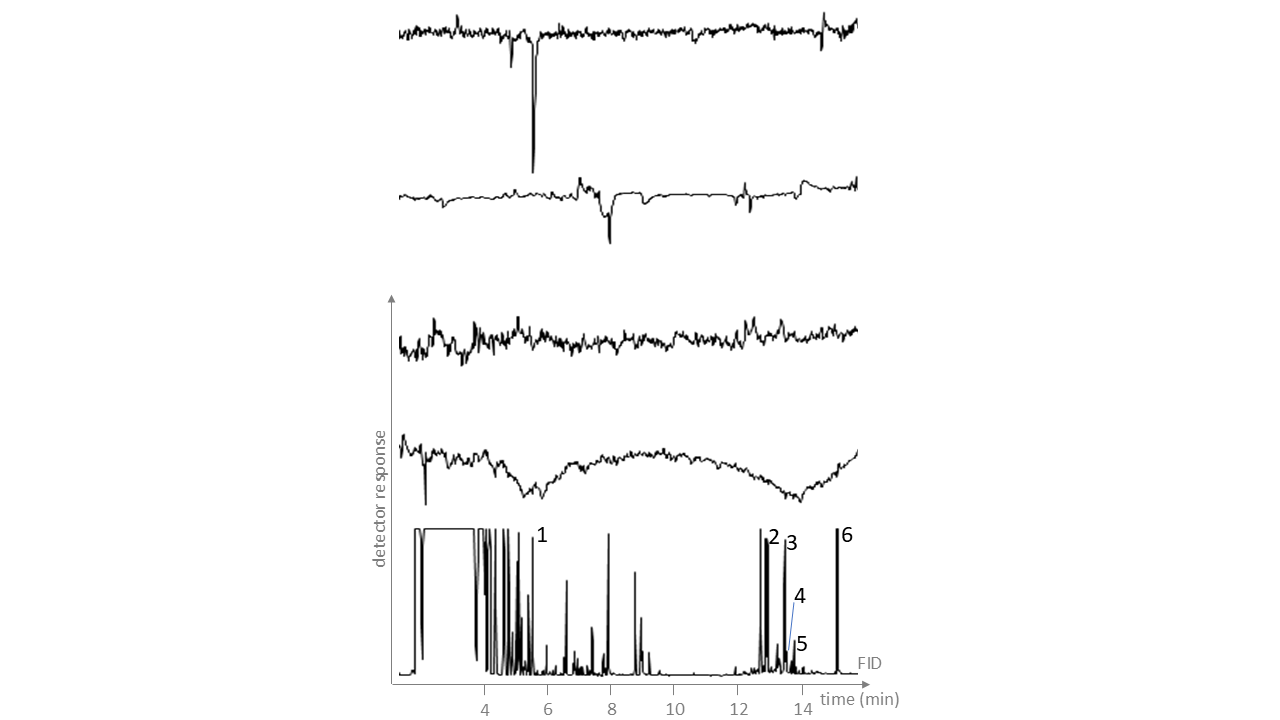


Figure S6. GC-EAD analysis of the WOND blend. Coupled GC-EAD traces with bioactive components: 1. hexanal, 2. (*E*)-caryophyllene, 3. (+)-δ-cadinene, 4. (*E,E*)-α-farnesene, 5. bisabolene, 6. n-heptadecane.
